# Operator Dependent Choice of Prostate Cancer Biopsy Has Limited Impact on a Gene Signature Analysis for the Highly Expressed Genes IGFBP3 and F3 in Prostate Cancer Epithelial Cells

**DOI:** 10.1371/journal.pone.0109610

**Published:** 2014-10-08

**Authors:** Zhuochun Peng, Karl Andersson, Johan Lindholm, Inger Bodin, Setia Pramana, Yudi Pawitan, Monica Nistér, Sten Nilsson, Chunde Li

**Affiliations:** 1 Department of Oncology-Pathology, Karolinska Institutet, Stockholm, Sweden; 2 Department of Oncology, Radiology and Radiation Sciences, Uppsala University, Uppsala, Sweden; 3 Clinical Pathology/Cytology, Karolinska University Hospital, Stockholm, Sweden; 4 Department of Medical Epidemiology and Biostatistics, Karolinska Institutet, Stockholm, Sweden; 5 Clinical Oncology, Karolinska University Hospital, Stockholm, Sweden; 6 Chundsell Medicals AB, Stockholm, Sweden; 7 Ridgeview Instruments AB, Uppsala, Sweden; 8 Sekolah Tinggi Ilmu Statistik/Institute of Statistics, Jakarta, Indonesia; University of Kentucky College of Medicine, United States of America

## Abstract

**Background:**

Predicting the prognosis of prostate cancer disease through gene expression analysis is receiving increasing interest. In many cases, such analyses are based on formalin-fixed, paraffin embedded (FFPE) core needle biopsy material on which Gleason grading for diagnosis has been conducted. Since each patient typically has multiple biopsy samples, and since Gleason grading is an operator dependent procedure known to be difficult, the impact of the operator's choice of biopsy was evaluated.

**Methods:**

Multiple biopsy samples from 43 patients were evaluated using a previously reported gene signature of IGFBP3, F3 and VGLL3 with potential prognostic value in estimating overall survival at diagnosis of prostate cancer. A four multiplex one-step qRT-PCR test kit, designed and optimized for measuring the signature in FFPE core needle biopsy samples was used. Concordance of gene expression levels between primary and secondary Gleason tumor patterns, as well as benign tissue specimens, was analyzed.

**Results:**

The gene expression levels of IGFBP3 and F3 in prostate cancer epithelial cell-containing tissue representing the primary and secondary Gleason patterns were high and consistent, while the low expressed VGLL3 showed more variation in its expression levels.

**Conclusion:**

The assessment of IGFBP3 and F3 gene expression levels in prostate cancer tissue is independent of Gleason patterns, meaning that the impact of operator's choice of biopsy is low.

## Introduction

Predicting the prognosis of cancer disease by using gene expression analysis is an approach reported in an increasing number of studies [1]. For many cancer diagnoses, a convenient and accessible sample type is formalin fixed paraffin embedded (FFPE) material from either biopsy material or surgically removed tumor tissue. For prostate cancer (PCa), FFPE biopsies are readily available in the clinical routine pathology laboratories and suitable for such analyses. However, in many cases multiple biopsies are available for each patient, and gene expression analysis is normally conducted on only one sample per patient. This means that the pathologist has to choose which biopsy to analyze.

The Gleason Score (GS) grading system is the dominant histopathological grading method for prostatic carcinoma around the world, both in research and in clinical routine. Gleason score is the sum of the Gleason grades of the most common and the second most common tumor patterns. GS is one of the most important clinical parameters for indicating prognosis of survival for prostate cancer patients. There is however a large grey zone in GS 7 in terms of survival differences: for example patients with GS 3+4 have a much better prognosis than patients with GS 4+3 [2]. It is a commonly noticed fact that the Gleason grading, even when evaluated by skilled pathologists, is an operator dependent method. This leads to risks for reporting non-concordant results on the same tumor material, in particular when discriminating 3+4 from 4+3 [3]. This type of operator dependency may have an impact on gene expression analysis.

A recent report from our laboratory showed that a gene expression signature of IGFBP3, F3 and VGLL3 could estimate prostate cancer patients' overall survival at the time of diagnosis [4]. The three genes were selected in a stepwise manner from a starting set of 641 stem cell gene predictors. Hence, this signature potentially captures stem cell propensity or ‘stemness’ of cancer cells independent of histopathological subtype [4]. It was evaluated on a Swedish cohort of 189 PCa patients diagnosed between 1986 and 2001 with nearly completed follow-up overall survival data. In this cohort, 78% of the patients were primarily treated with hormone therapy only. The gene expression signature was shown sufficient to categorize the patients into high-risk, intermediate-risk and low-risk subtypes.

The IGFBP3, F3 and VGLL3 gene signature was identified using Fine Needle Aspiration (FNA) cytology samples. The current clinical practice for prostate cancer diagnosis is to use FFPE core needle biopsy samples. An advantage of FFPE samples is that they can be easily archived and that many cohorts have long time follow-up clinical data available, which greatly facilitates clinical studies. Even though the extracted RNA from FFPE samples may be of relatively low quality, multiple recent studies have shown promising results when utilizing degraded RNA extracted from archival FFPE samples for quantifying gene expression levels by optimized qRT-PCR methods [5]–[7]. One example is the Prostatype qRT-PCR kit, which is developed and optimized for measuring the gene expression levels of a gene signature of IGFBP3, F3 and VGLL3 particularly in FFPE samples.

In the analysis of RNA expression levels in FFPE biopsy samples, there are a multitude of factors that have an impact on the results. Each biopsy has a different fraction of tumor material and the storage conditions may have influenced RNA quality in different ways. To that, there is a number of factors in the analytical procedure that will induce variation, such that RNA extraction efficiency, pipetting errors, reagent stability over time, impurities, contamination and so on.

In the light of the variability and operator dependency of Gleason score ranking, we investigated the expression level of IGFBP3, F3 and VGLL3 in multiple biopsies from the same patient, and focused on the variation related to the very choice of which biopsy to be analyzed. This made it possible to assess the impact of the operator's choice of sample on the measured gene expression level.

## Materials and Methods

### Ethics statement

The study protocol was approved by the Research Ethics Committee of the Karolinska University Hospital (approval number: 00-164) and was performed in accordance with the ethical principles described in the 1964 Declaration of Helsinki.

Informed written consent for general bio-bank material collection was obtained from all participants according to the Swedish bio-bank law, prior to their inclusion in the study.

### FFPE prostate core needle biopsies

The FFPE prostate core needle biopsy samples were collected at the time of PCa diagnosis according to the routine procedure used at Aleris Diagnostik AB (Aleris Medilab) in Stockholm, Sweden. For each patient, 6–10 core needle biopsies were taken for Gleason grading [2]. Biopsies from 43 patients diagnosed with PCa between 2004–2007 where used for this study. Until the end of 2013, 22 of these patients had died of PCa, 10 had died of other diseases and 11 were still alive. All biopsies had been stored in the Aleris Medilab biobank facility at conditions suitable for FFPE samples for at least 6 years before use in this analysis.

### Sample preparation and RNA extraction

The sample preparation process prior to RNA extraction is described step by step as follows.

#### Step a: Sectioning of FFPE samples

FFPE core needle biopsies were cut into separate sections and were attached onto frosted glass slides essentially according to routine histopathological protocols [8], except that DNase/RNase free water was used and the step of melting paraffin after drying sections was omitted. The first qualified section of 5 µm thickness was H&E stained [9], and used for marking the cancer region by pathologists. Sequential sections of 10 µm thickness after the first H&E stained section, were collected onto glass slides without DNase/RNase contamination and used for RNA extraction.

#### Step b: Marking and quantifying cancer area

A digital pathology slide scanner (iScan Coreo, Ventana Medical Systems, Inc.) was used for image-based documentation of the marked cancer region to be scraped and analyzed. First, the original H&E stained slide generated in step (a) was digitally scanned. The scanned image was magnified up to 20 times, the cancer area was marked, and calculated using image viewing software (Image Viewer, Ventana Medical Systems, Inc.). Furthermore, the first and second Gleason patterns were noted. Then the cancer area marks were copied from the image and marked back onto the original H&E stained glass slide using a microscope, serving as a ‘map’ that guided the cancer cells scraping procedure.

#### Step c: Scraping cancer area

Using the marked H&E stained glass slide as a map, the corresponding areas of the unstained 10 µm-thick FFPE sections were identified. For each Gleason pattern, an area of at least 10 mm^2^ cancer cell containing tissue was scraped into DNase/RNase free micro centrifuge tubes using a new disposable scalpel for each sample to be extracted. The majority of scraped samples contained more than 70% cancer epithelial cells.

### Sample measurements

For each sample measurement, tumor material from one type of pathological pattern (Gleason tumor pattern or benign pattern) was collected, sometimes from different biopsies from the same patient, into one vial for further analysis. This means that the pathologists collected cancer areas with the same histopathological type of cells from one or more biopsies to harvest sufficient tissue for the analytical procedure. The percentage of tumor material in each cancer cell containing sample was evaluated and confirmed by pathologists using digitally scanned images of H&E stained slides. We define a cancer sample as a measurement on a tumor sample with a large fraction of cancer cells, and a benign sample (from the same prostate cancer patient included in the study) as a measurement on a prostate tissue sample containing benign cells only.

In total, 127 valid sample measurements derived from 43 patients were produced. For these 43 patients, we measured 97 cancer samples. For 30 of the 43 patients, one benign sample per patient was collected and measured, in addition to their cancer samples (Table 1).

**Table 1:** Characteristics of samples and patients.

Patient sample measurements^a^:	Patients, n (%)	Different/Similar Cancer Samples (Total Cancer Samples)^b^, n	Cancer/Benign Samples (Total samples)^c^, n
Patients with two cancer samples	33 (76.7)	62/4 (66)	66/27 (93)
Patients with three cancer samples	9 (20.9)	27/0 (27)	27/3 (30)
Patients with four cancer samples	1 (2.3)	4/0 (4)	4/0 (4)
Total	43 (100)	93/4 (97)	97/30 (127)
Mortality:	Patients, n (%)		
Diagnosed with prostate cancer	43 (100)		
Death due to prostate cancer	22 (51.2)		
Death due to other causes	10 (23.3)		
Alive after 5 years	11 (25.6)		
Gleason score:	Patients, n (%)		
3+3	1 (2.3)		
3+4	9 (20.9)		
4+3	9 (20.9)		
4+4	4 (9.3)		
4+5	8 (18.6)		
5+4	10 (23.3)		
5+5	2 (4.7)		
Total	43 (100)		
Tumor percentage^d^:	Primary/Secondary cancer samples, n (%)		
>90%	77 (79.4)		
>80%	5 (5.2)		
>70%	9 (9.3)		
>60%	3 (3.1)		
>50%	3 (3.1)		
Total	97 (100)		

a Each sample contains a pathological type of cells. Each patient had been measured with one primary cancer sample and at least one secondary cancer sample, referring to the 1^st^ and 2^nd^ most common Gleason pattern.

b Similar cancer samples refer that patients had very similar cancer samples with respect to the Gleason pattern.

c A majority of patients also had one benign sample measurement besides their primary and secondary cancer sample measurements.

d Cancer cells herein represent cancer epithelial cell. The tumor percentage of each cancer cell containing sample was evaluated and confirmed by pathologists using H&E slides or digitally scanned images.

Among the cancer sample measurements, 33 of the 43 patients had two cancer samples were evaluated, the remaining patients had three or four cancer samples evaluated. From 41 patients there was one primary cancer sample and at least one secondary cancer sample available. The remaining 2 patients had two very similar primary cancer samples with respect to the Gleason pattern (Table 1).

### Total RNA extraction

Total RNA was extracted from scraped tissue samples using the High Pure FFPE RNA Micro Kit (Roche Applied Science/Roche Diagnostics GmbH, catalog number: 4823125001) according to vendor's instruction. Extracted RNA was immediately subjected to the qRT-PCR analysis.

### One-Step qRT-PCR reaction

Expression levels of IGFBP3, F3, VGLL3 and GAPDH were measured using a pre-production version of the commercial Prostatype qRT-PCR kit, (Chundsell Medicals AB, Sweden). This is a four-multiplex one-step qRT-PCR kit, which is custom designed to measure gene expression levels of the three biomarker genes IGFBP3, F3, and VGLL3, normalized to the expression level of the gene GAPDH (glyceraldehyde 3-phosphate dehydrogenase) in RNA extracted from FFPE human prostate cancer tissue samples. The sequence information of probes and primers has been reported previously [4]. Extracted total RNA can be used for this qRT-PCR reaction immediately even without quantifying the input of RNA according to the instructions for use. The kit further contains positive and negative controls, which were assayed together with each batch of prostate cancer tissue samples. Measurements were conducted using Roche LightCycler 480 instrument II, a qPCR platform on which a color compensation method was run prior to performing the qPCR analysis.

### Data analysis

Ct values of all batches of samples were extracted according to instructions from the manufacturers (Roche and Chundsell Medicals). A batch of qRT-PCR experiments was considered valid only if positive and negative controls were valid. Samples with GAPDH Ct value of ≥29.0 were excluded due to low amount of extracted RNA. The expression levels of the IGFBP3, F3, and VGLL3 genes were normalized to that of GAPDH and were presented as the delta Ct value, which is inversely correlated to the gene expression level. These delta Ct values were used for a statistical analysis of the positive control sample performance. Delta Ct values were further used to form Diff (delta Ct)  =  max (delta Ct value)-min (delta Ct value) for two different tumor samples obtained from the same patient. Histograms and scatterplots were used in the analysis of Diff (delta Ct).

## Results

For the majority of patients (76.7%), two different cancer samples were evaluated. The remaining patients had three or four different cancer samples evaluated (Table 1). Most of the patients were tested in 2 sample measurements, each containing two Gleason patterns. The distribution of patients according to the Gleason score shows that more than 80% of the patients had odd Gleason scores, such as 3+4/4+3 (7) or 4+5/5+4 (9). In the majority (79.4%) of the cancer sample measurements of primary and secondary tumor patterns, the tissue material contained more than 90% cancer epithelial cells. The remaining measurements of primary and secondary tumor patterns had larger proportions of stromal cells, in a few cases even more than 40% stromal cells.

Variations in expression levels of the three signature genes (IGFBP3, F3, and VGLL3) across multiple cancer samples from the same patient, particularly in those cancer sample measurements that represented the first and the second Gleason patterns, were used to assess the operator's impact on the reported result. The differences in delta Ct values of the signature genes from sample measurements taken from the same patient were used to characterize gene expression level variability. For example, for a certain patient, difference of delta Ct value of F3 equals delta Ct F3 from sample one minus delta Ct F3 from sample two. Delta Ct values for primary cancer samples are shown in Table S1.

**Figure 1:**
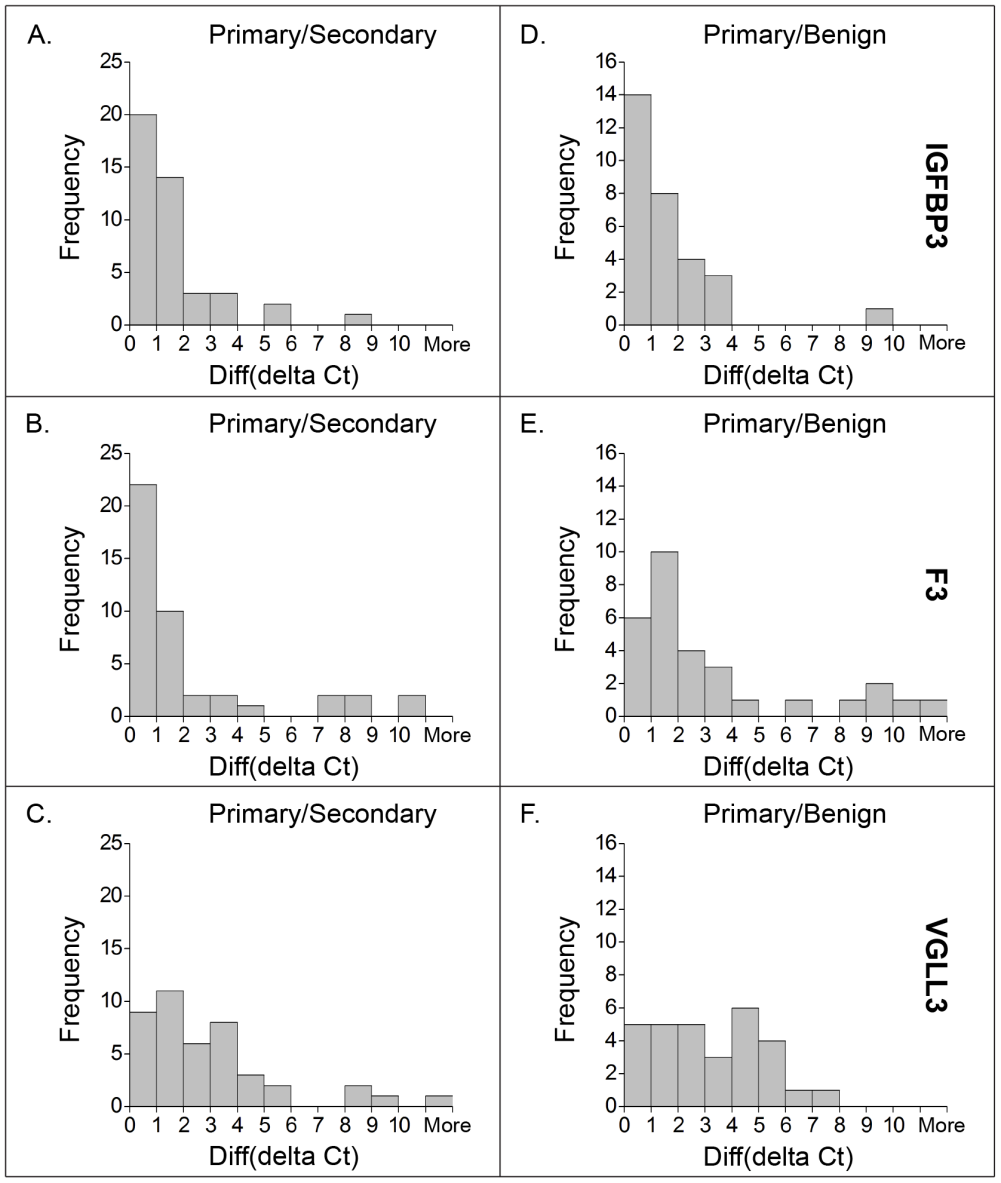
Comparisons of differences in gene expression levels within primary/secondary cancer samples and primary/benign samples. The same definition of sample measurements as described in Materials and Methods section is used here. The expression levels of three genes were measured as the delta Ct values (i.e. Ct value of gene-Ct value of GAPDH). The differences in gene expression levels between different samples originating from the same patient are presented as Diff (delta Ct),  =  max (delta Ct value)-min (delta Ct value). We estimated the similarity of gene expression levels in tumor biopsies by comparing the three genes' Diff (delta Ct) values in primary and secondary cancer samples from 43 patients (Panels A, B and C) and presenting results in the form of frequency histograms. Among these patients, 30 patients were also measured for gene expression in their accompanying benign prostate tissue samples, and results are presented in the right panels (Panels D, E and F), as frequency histograms of Diff (delta Ct) values between the primary cancer samples and benign samples. The frequency counts for delta Ct value differences within 0–1.0 or 1.0–2.0 interval were dramatically higher than those with differences larger than 2.0 for IGFBP3 and F3 as shown in the left panels of comparison. Compared to the left panels, the Diff (delta Ct) values in the right panels showed higher frequency in the larger Diff (delta Ct) value intervals for all three genes, but particularly for VGLL3.

In order to illustrate the distribution of the delta Ct value differences (Diff (delta Ct)), we generated frequency histograms of differences for each of the three genes' expression levels obtained in sample measurements for a certain patient (Figure 1). A histogram represents tabulated frequencies, shown as rectangles, erected over discrete intervals (i.e. Diff (delta Ct)), each with an area proportional to the frequency of the observations in that interval. In this context, with an increase of 1.0 in Diff (delta Ct) for each interval, the frequency of observations was counted within each interval, equals the area of a rectangle.

Figure 1A, B, C shows histograms of Diff (delta Ct) for the three different genes when comparing primary and secondary Gleason tumor patterns. For IGFBP3, 34 out of 43 patients (79%), the Diff (delta Ct) within measurements was less than 2.0. For F3, 32 of 43 (74%) patients had Diff (delta Ct) less than 2.0. For VGLL3, only 20 (46.5%) patients had Diff (delta Ct) ≤2.0, 14 (32.5%) patients had Diff (delta Ct) values in the range 2.0–4.0 and the remaining 9 patients had differences in delta Ct value exceeding 4. It was further observed that the mean Ct values of IGFBP3, F3 and VGLL3 of all 97 cancer sample measurements were 28.7, 28.3 and 33.2 respectively, meaning that VGLL3 generally had lower expression levels compared to IGFBP3 and F3 in prostate cancer samples.

In parallel with the sample measurements, the positive control sample was measured 40 times. For IGFBP3 positive control measurements, the reported delta Ct value was in average 3.68, the standard deviation was 0.42, and the max/min values were 4.37/2.62. For F3, the corresponding values were 1.87; 0.37; 2.61/0.71, and for VGLL3, they were 5.12; 0.36; 5.91/3.76. Based on this, the typical variation of delta Ct values from run to run due to the assay as such was estimated to be two standard deviations, i.e. 0.84 for IGFBP3, 0.74 for F3 and 0.72 for VGLL3.

Figure 1D, E, F shows histograms of Diff (delta Ct) for the three signature genes when comparing primary tumor pattern with benign tissue. In this comparison, IGFBP3 consistently provided values similar to the Diff (delta Ct) within the primary and secondary cancer samples from each patient (Figure 1D). In fact, the primary pattern was almost equally similar to the benign pattern as to the secondary pattern. For F3, the primary-benign similarity was less than the primary-secondary similarity (Figure 1E). VGLL3 showed large Diff (delta Ct) discrepancies between the primary Gleason tumor pattern and the benign sample (Figure 1F).

**Figure 2:**
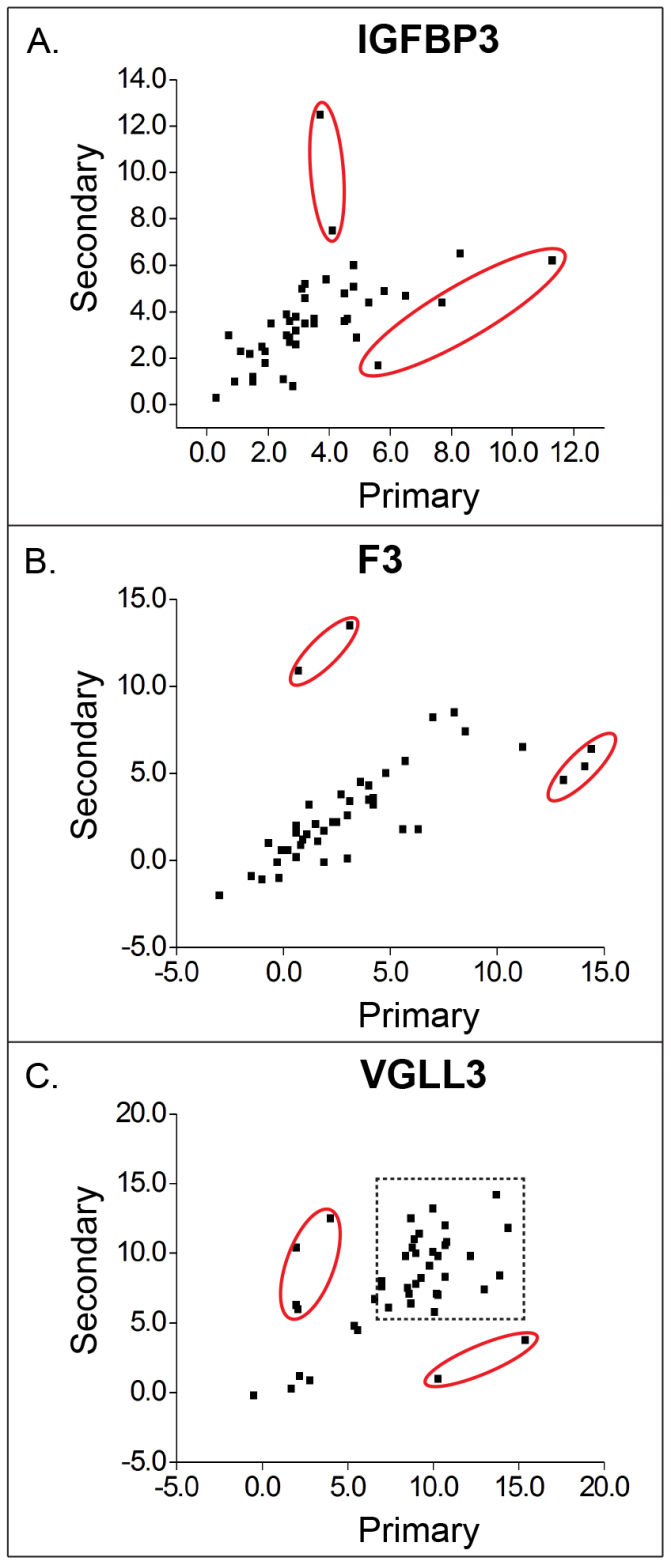
Scatterplots of gene expression levels derived from the primary and the secondary cancer sample measurements. In order to measure how similar the primary and secondary cancer samples were in terms of gene expression levels, and further for the outlier analysis, we generated scatterplots of gene expression levels of the two measurements. The expression levels of the three signature genes are shown as delta Ct values using the same equation as described in the legend of Figure 1. The primary and secondary cancer sample measurements refer to the two measurements containing the first and second most common Gleason patterns. The two types of cancer samples from 43 patients are compared using scatterplots. Red circles indicate the outliers, which were further investigated regarding tissue input quantity and percentages of cancer epithelial cells. Dashed line square indicates VGLL3 measurements that had high delta Ct values and poor reproducibility.

An alternative way of illustrating the differences in gene expression between primary and secondary tumor patterns is to make scatterplots of delta Ct values from measurements of these cancer samples (Figure 2). In this view, it is clear that comparability is good, in particular for low delta Ct values, with the exception of a few outliers (red circles). The lower degree of comparability for VGLL3 is mainly due to differences at high delta Ct values meaning relatively low expression levels (dashed line square).

The outliers in the scatterplots of the three genes in Figure 2 (red circles) were further investigated by checking the tissue input, the percentages of cancer cells of marked areas or the special histopathological patterns of the selected cancer areas. The majority of the outliers were obtained from samples that had one of the following properties: <0.1 mm^3^ tissue input, containing >30% stromal cells or representing infiltrative/invasive cancer type, within which cancer cells usually are encapsulated by stromal cells. This tissue type was found in 4 of 5 outliers for IGFBP3, for F3 3 of 5 and for VGLL3 4 of 6. One patient produced outliers in all three comparisons, and another patient produced outliers for both F3 and VGLL3. Remaining outliers could not be explained.

## Discussion

This work evaluates the operator's impact on the choice of which biopsy to be used for gene expression analysis. In order to minimize the overall variation of test results in gene expression assays, it is important to assess the variability induced by subjective decisions and thereby the possibility to reduce the variation through improved practical procedures. In fact, this study of a three genes expression signature in prostate cancer included a large proportion (80%) of patients with odd Gleason score such as 3+4/4+3 (i.e. 7) or 4+5/5+4 (i.e. 9), where we consider the risk for operator mistakes to be largest.

The IGFBP3 and F3 genes showed limited differences in expression levels within cancer epithelial cell samples, when comparing delta Ct values from different biopsies originating from the same patient. Within cancer tissue measurements representing different histopathologically determined Gleason patterns, the typical variation was 1–2 delta Ct value units. When comparing a biopsy with benign cells with that of the primary Gleason pattern, a similar variation was observed for IGFBP3 and slightly larger variation for F3. Since the positive control variation was 0.7–0.8 delta Ct value units, the choice of biopsy is a source of error and variation of similar magnitude as the assay itself.

The comparison of delta Ct values from tissue samples originating from the same patient can be conducted in many ways. With access to multiple biopsies from only 43 patients, the number of suitable methods for comparing output is unfortunately limited. We have chosen to use histograms to present the differences, because this provides a clear illustration of the distribution of differences. For example, in Figure 1A it is evident that the majority of the delta Ct differences when comparing primary and secondary cancer sample is between 0 and 2 delta Ct units, accompanied by a few outliers. Any attempt to estimate concordance by using statistical methods would be heavily biased by the outliers, hence the choice of histogram representation of the results, accompanied by scatterplots.

Measurements of VGLL3 expression levels showed larger variation between biopsies, which mainly was caused by a low expression level in prostate cancer cells compared to the other two signature genes. The average delta Ct of VGLL3 in positive control assays showed the probe and primers for VGLL3 are adequately functional, meaning that the expression level of VGLL3 is truly low in prostate tissue. Accurate quantification of such low expression levels can probably be improved by increasing the input of cancer tissue material so as to enhance the signal. Other factors that can influence variability of VGLL3 measurements are variations in stromal cell content in the different biopsy samples or non-uniform mRNA degradation, meaning that VGLL3 might suffer from more extensive degradation than the other tested genes or might be differently distributed among epithelial and various stromal cells.

Measurements of the gene signature in benign tissue revealed that IGFBP3 consistently provided similar values to those in the tumor tissue from the same patient, F3 remained similar but with larger variation, and VGLL3 was highly variable. Since the cancer tissues used for measurement contained varying levels of stromal cells, this type of analysis is important to learn how to guide the operator in biopsy selection. In gene expression measurements of both IGFBP3 and F3, the primary and secondary tumor patterns provided results that were in agreement, and for these cases it was known that only a minor fraction of the tumor samples in fact contained stromal cells. Since F3 lost parts of the expression level comparability towards benign tissue only, it is important to have a large fraction of tumor cells in the biopsy sample used for gene expression analysis. Our current best estimate is that approximately 2/3 of the tissue material should contain cancer epithelial cells for F3 to produce comparable results. For VGLL3, the comparability is poor, but it is unknown if this is related to this gene being differently expressed in tumor contra benign tissue, in epithelial cancer cells versus stromal cells or if this is simply an effect of the technical difficulties in measuring low expression levels.

An important observation is that the impact of the operator's choice of which biopsy is the primary in terms of tumor pattern (Gleason pattern) is not a dominant source of error for the two genes that were highly expressed, i.e. IGFBP3 and F3. This means that a gene expression analysis using IGFBP3 and F3 will be insensitive to moderate differences of the Gleason grading. Furthermore, for IGFBP3 and F3 the procedure is insensitive to the fraction of stromal cells, as long as approximately 2/3 of the tissue material is cancer cells. These two aspects are important factors that contribute to a truly objective gene expression assessment of the patient, which when combined with traditional clinical parameters like Gleason score may contribute to more accurate prognostic statements. Another important observation is that since the expression analysis of the samples representing the primary and secondary patterns is similar for IGFBP3 and F3, there is no need to keep them separated, but all tumor material from a single biopsy can most likely be collected into one vial for gene expression analysis. For VGLL3, the most observed large differences occurred at low expression levels. Only after the analytical procedure has been altered to handle such low amounts, the performance profile for VGLL3 across a variety of tumor patterns can be investigated.

Often when a prognostic or diagnostic gene expression panel has been developed on large and fresh (or fresh frozen) tissue material from surgical samples the challenge will lay in transferring the assay to handle samples, which are available in clinical routine, in particular using biopsy samples containing small cancer areas. FFPE tissue sections are common in routine cancer diagnostics, but contain lower-quality DNA/RNA due to the harsh tissue preparation steps and long-term storage. This report illustrates that for the two highly expressed genes, the choice of which tissue specimen to analyze has small (if any) impact on the analysis result, which is encouraging for the general case of transferring gene expression assays from fresh tissue material to FFPE material.

For the purpose of using the IGFBP3, F3, VGLL3 gene expression profile for providing supplementary information on the prognosis of individual patient's prostate cancer disease, valid assay data is required. This report concludes that the operator dependency will not be a dominant source of error for IGFBP3 and F3 in this gene expression profile, which is of critical importance for the practical implementation of the gene expression assay in routine use. The gene expression profile is currently evaluated on prostate cancer patient's material for it's ability to aid in making prognostic decisions, and the results of that study will be reported at a later time.

Should the finding reported in this report prove general, the supporting evidence for the underlying ‘stemness’ hypothesis in initiation and progression of (prostate) cancer would be strengthened. As the gene expression levels of IGFBP3 and F3 were independent of histopathological grading in prostate cancer area, the same as in benign area, the gene signature may reflect underlying pathogenetic mechanisms of prostate cancer. Based on this observation, the assay transfers would have a high success-rate in clinical practice.

In summary, we report that the operator's choice of which biopsy to conduct gene expression analysis on does not have any dominant impact on the results of IGFBP3 and F3 gene expression measurements in cancer epithelial cells. The analysis of the VGLL3 gene expression levels would benefit from optimization due to generally low expression levels.

## Supporting Information

Table S1 **Delta Ct values indicating the three genes expression levels in the primary cancer samples of 43 patients.**
(DOCX)
