# Supplementary material for: Operator Dependent Choice of Prostate Cancer Biopsy Has Limited Impact on a Gene Signature Analysis for the Highly Expressed Genes IGFBP3 and F3 in Prostate Cancer Epithelial Cells
Source: PLoS One. 2014 Oct 8;9(10):e109610. doi: 10.1371/journal.pone.0109610 (PMC4190108; doi:10.1371/journal.pone.0109610)
Supplement: Table S1 — Delta Ct values indicating the three genes expression levels in the primary cancer samples of 43 patients. (DOCX) [file pone.0109610.s001.docx]

Table S1. Delta Ct values indicating the three genes expression levels in the primary cancer samples of 43 patients^a^.

|  | Delta Ct IGFBP3 | Delta Ct F3 | Delta Ct VGLL3 |
| --- | --- | --- | --- |
| PT30 | 1.92 | 1.61 | 10.82 |
| PT137 | 3.54 | 0.78 | 9.78 |
| PT12 | 1.53 | 3.98 | 6.55 |
| PT7 | 1.54 | 3.99 | 8.54 |
| PT79 | 3.18 | 0.2 | 9.32 |
| PT15 | 2.57 | 2.45 | 1.68 |
| PT8 | 0.3 | -0.98 | 5.35 |
| PT133 | 3.23 | 5.66 | 2.22 |
| PT106 | 8.33 | 1.94 | 10.32 |
| PT180 | 4.46 | 1.09 | 8.56 |
| PT155 | 2.56 | 3.57 | 13.68 |
| PT37 | 1.88 | 4.22 | 7.39 |
| PT169 | 1.13 | 0.86 | 8.42 |
| PT27 | 2.69 | 8.04 | 8.92 |
| PT31 | 0.72 | -3.01 | 9.95 |
| PT74 | 3.24 | -1.49 | 8.95 |
| PT1 | 2.65 | 1.87 | 8.97 |
| PT160 | 4.47 | -0.21 | 9.97 |
| PT5 | 0.85 | 4.83 | 7.03 |
| PT24 | 2.87 | 1.49 | 10.15 |
| PT23 | 1.8 | 2.98 | 2.01 |
| PT63 | 2.05 | -0.7 | 9.15 |
| PT109 | 7.66 | -0.26 | 12.17 |
| PT172 | 5.8 | 3.07 | 2.78 |
| PT94 | 4.06 | 7.04 | 10.65 |
| PT34 | 2.81 | 2.29 | 10.09 |
| PT35 | 2.45 | 3.02 | 10.71 |
| PT66 | 4.61 | 4.15 | 6.97 |
| PT70 | 5.32 | 1.15 | 5.59 |
| PT105 | 3.5 | 13.14 | 10.68 |
| PT96 | 1.4 | 14.35 | -0.46 |
| PT162 | 4.75 | 0.64 | 4.03 |
| PT25 | 5.57 | 8.45 | 13.85 |
| PT117 | 2.86 | 5.58 | 12.99 |
| PT20 | 2.7 | -0.13 | 14.39 |
| PT89 | 4.76 | 0.74 | 8.7 |
| PT43 | 6.45 | 0.61 | 10.28 |
| PT52 | 2.91 | 0.64 | 15.36 |
| PT88 | 3.93 | 3.07 | 8.77 |
| PT26 | 3.68 | 2.7 | 8.7 |
| PT38 | 3.12 | 11.17 | 10.31 |
| PT112 | 4.85 | 14.14 | 2.12 |
| PT81 | 11.33 | 6.27 | 2.01 |

^a^Primary cancer sample contains a large fraction of one pathological type of cells referring to the 1^st^ most common Gleason pattern.
